# The Role of Free Radicals in the Mimetic Naphthalene Dioxygenase Function of *Trametes versicolor* Laccase Application for Biosynthesis of Isatin and Indirubin

**DOI:** 10.3390/molecules31132352

**Published:** 2026-07-03

**Authors:** Lin Wang, Tingting Wang, Yunqin Liu, Boya Zhang, Tao Meng, Xuecai Luo, Zai Zheng, Yun Zhang, Ruoshui Wang, Ming Chen, Jihu Su, Liang Zhang, Ruochun Yin

**Affiliations:** 1 School of Life Sciences and Medical Engineering, Anhui University, Hefei 230039, China; d24301082@stu.ahu.edu.cn (T.W.); d24301094@stu.ahu.edu.cn (Y.L.); d125221124@stu.ahu.edu.cn (B.Z.); mengtao5883@163.com (T.M.); 15855120227@139.com (X.L.); d72214041@stu.ahu.edu.cn (Y.Z.); 2 National Key Laboratory of Tropical Crop Breeding, Institute of Tropical Bioscience and Biotechnology, Chinese Academy of Tropical Agricultural Sciences, Haikou 571101, China; zhengzai@catasitbb.cn; 3 School of Biotechnology, Jiangnan University, Wuxi 214122, China; 1020325121@stu.jiangnan.edu.cn (R.W.); zhangl@jiangnan.edu.cn (L.Z.); 4 CAS Key Laboratory of Microscale Magnetic Resonance, Department of Modern Physics, University of Science and Technology of China, Hefei 230026, China; chen5566@ustc.edu.cn (M.C.); sujihu@ustc.edu.cn (J.S.)

**Keywords:** *Trametes versicolor*, laccase, Naphthalene, 1,2-naphthalenediol, dioxygenation

## Abstract

Naphthalene (Nap) is widely distributed in the environment and has attracted concern due to its recalcitrance to biodegradation and acute toxic effects. Although several laccases can degrade naphthalene, it remains unclear whether laccase is involved in dioxygenation or ring fission of polycyclic aromatic hydrocarbons (PAHs). We demonstrated that laccase has a naphthalene dioxygenase function with a distinct joint approach to oxygen and the alternative regioselective ring-fission process. These results supplement the family of dioxygenase with laccase, a mild method for the ring opening of aromatics, and an operable scale-up application for remediation from PAH pollution and for regioselective oxygenation and ring opening.

## 1. Introduction

Edible mushrooms were widely consumed as nutritious foods due to their high-quality protein and bioactive components that contribute to human health. In commercial cultivation, edible fungi were typically grown on agricultural and forestry by-products, which provides a cost-effective production strategy. However, these substrates might contain contaminants such as biotoxins, pesticides, and antibiotic residues [1]. Strangely, previous studies rarely reported that pollutants’ detection in mushroom fruiting bodies, suggesting that edible fungi may possess intrinsic mechanisms for degradation or transformation of toxic compounds during growth [2,3]. These observations indicate that understanding how edible fungi detoxify or utilize such contaminants needs exploration.

*Trametes versicolor* is a traditional medicinal mushroom whose bioactive constituents possess antitumor, antioxidant, hypoglycemic, and immunomodulatory properties and have been widely utilized in food and pharmaceutical applications [4,5,6]. Among the enzymes produced by *T. versicolor*, laccase, a member of the lignin oxidase family, belongs to multicopper oxidases widely distributed in bacteria, fungi, green plants, and insects [7,8,9,10,11]. The active center of laccase consists of four copper ions organized into T1 and T2/T3 sites, enabling single-electron oxidation of a broad range of substrates and making laccase a versatile catalyst in organic synthesis and environmental remediation [12,13,14,15,16,17]. In particular, laccase participated in lignin degradation and the transformation of aromatic compounds, including organic pollutants such as polycyclic aromatic hydrocarbons (PAHs), suggesting its potential involvement in detoxifying environmental contaminants present in cultivation substrates.

PAHs, composed of two or more fused benzene rings, are widely distributed, persistent, and highly polluting environmental contaminants, whose microbial degradation requires the synergistic action of multiple oxidases through complementary mono- and dioxygenase pathways to accomplish oxidation and ring cleavage [18,19,20,21]. Naphthalene dioxygenases (NDOs), which act on PAHs, are well-documented to belong to the Cupin superfamily, exhibit a conserved histidine–aspartate–histidine (His-Asp-His) triad coordination motif, and catalyze the cleavage or ring opening of substrate double bonds [22,23,24,25,26]. Biocatalytic detoxification using oxidative enzymes has emerged as a promising strategy for mitigating PAH contamination under mild conditions. However, little evidence has been reported on laccase-mediated naphthalene (Nap) transformation, nor on its dioxygenase activity toward naphthalene.

NDO catalyzes the radical-mediated oxidative dearomatization of aromatic hydrocarbons, and insights into radical mechanisms and the modulation of reaction barriers and tunneling by enzyme microenvironments in analogous oxidases provide a core theoretical basis for understanding laccase-mediated NDO-like dioxygenase activity [27,28,29]. So-called white-rot fungi can oxidize PAHs in the presence of laccase and release hydroxyl free radicals [30]. Oxygenation was observed, and laccase offered a regioselectivity of PAH-quinones and acids rather than dihydrodiols that dioxygenases triggered products [8,31,32]. Oxygen activation and catalytic production of oxygenases have been well-established [33,34,35,36,37,38,39,40,41]. In contrast, the mechanism by which laccase causes PAH degradation remains uncertain, except that it is not involved in the initial oxygen insertion and ring fission of PAHs [16,17,42]. The previous studies showed that laccase propelled catechol and guaiacol derivatives to ring fission [43,44,45]. Therefore, the question of whether PAHs without side chains obey similar processes is important for microbial metabolism and regioselective synthesis. To solve the riddle, we focus on using high-performance liquid chromatography-mass spectrometry (LC-MS) and nuclear magnetic resonance (NMR) to monitor the intermediates. The process was analyzed using electron paramagnetic resonance (EPR) to identify the types of free radicals involved.

In this study, we report for the first time that laccase exhibits dioxygenation activity toward naphthalene, along with the ability to selectively catalyze ring opening at specific sites via free radical-mediated mechanisms. These properties of laccase will provide a new catalytic strategy for constructing regio/enantioselective aromatic ring transformation systems in the fields of organic chemistry and compound synthesis, while understanding this mechanism is crucial for developing enzyme-based strategies for the mitigation of PAH contamination in food-related.

## 2. Results and Discussion

### 2.1. Confirmation of Laccase’s Dioxygenase Function

**Figure 1:**
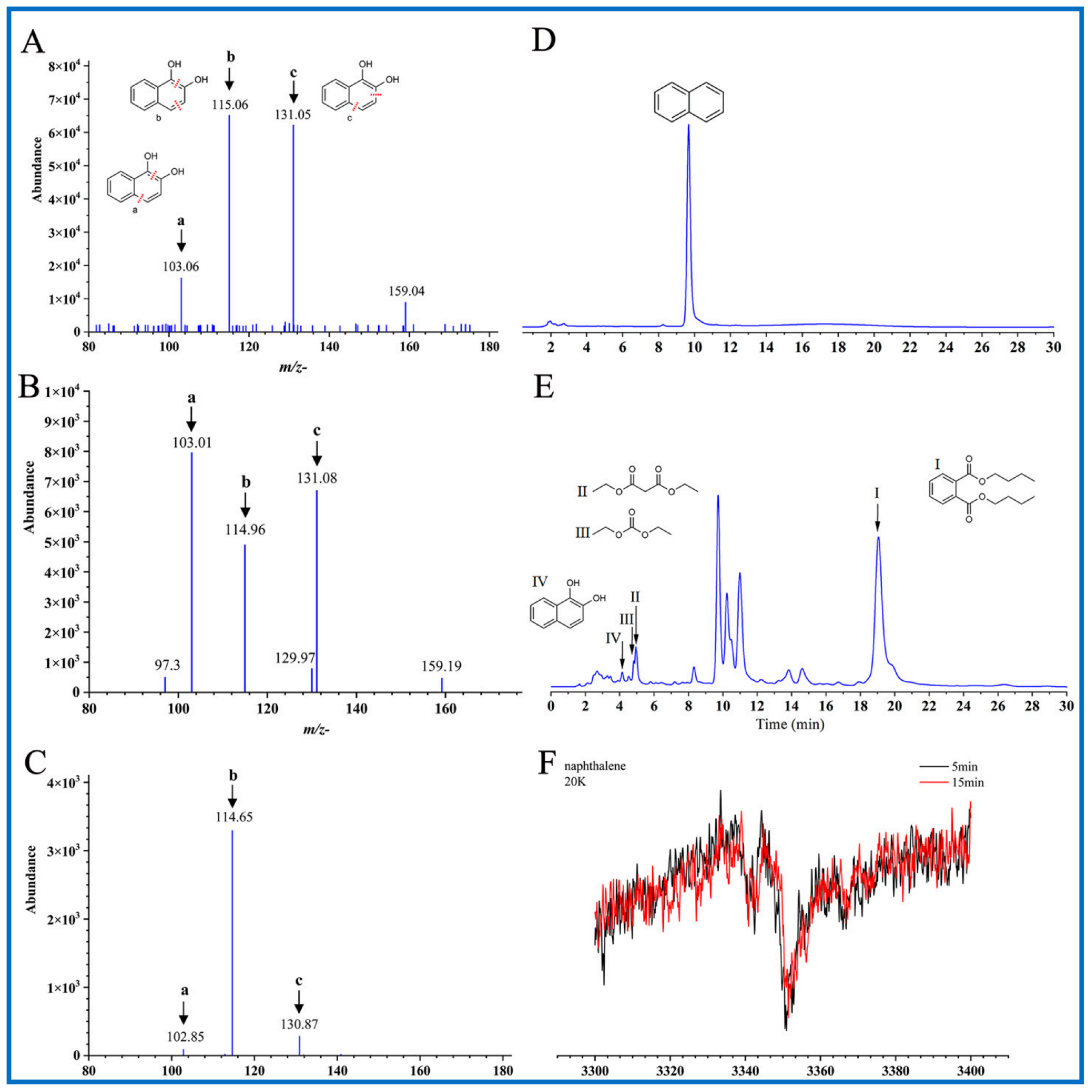
Tandem mass spectrometry (MS-MS) spectrum of 1,2-naphthalenediol (1,2-DHN) standard and catalysts with the same *m/z*^−^ of 159.05, High-performance liquid chromatography (HPLC) spectrum of naphthalene (Nap) and catalysts and electron paramagnetic resonance (EPR) spectra of the laccase-catalyzed oxidation of naphthalene trapped by freezing trapping. (**A**) MS-MS spectrum of 1,2-naphthalenediol standard. (**B**) The MS-MS spectrum of the peak with the same retention time, 3.96 min, from the filtrated reaction mixture in H_2_^16^O aqueous solution. (**C**) The MS-MS spectrum of the peak with the same retention time, 3.96 min, from the filtrated reaction mixture in H_2_^18^O aqueous solution. The MS data were obtained by LC-MS online analysis as described in Experimental Procedures. Both compounds showed the same *m/z*^−^ as the 1,2-naphthalenediol standard and generated nearly identical fragments (*m/z*^−^ 103, 115, and 131). The *m/z*^−^ 103, 115, and 131 ions fit the fragmentation of 1,2-naphthalenediol at a, b, and c respectively (inset). (**D**) Naphthalene standard (2.3 mM) analyzed by HPLC. (**E**) Naphthalene incubated with laccase for 72 h analyzed by HPLC. All traces were from a 254 nm UV profile. (**F**) EPR spectra were recorded to monitor the laccase-catalyzed oxidation of naphthalene trapped by freezing trapping. The reaction proceeded at room temperature for 5 and 15 min, and the solutions were stored quickly in liquid helium until EPR measurements.

The intermediates and oxygen atom sources were determined in the laccase-catalyzed naphthalene oxidation. LC-MS analysis showed a new peak with an identical retention time to the 1,2-naphthalenediol (1,2-DHN) standard when naphthalene was subjected to catalytic oxidation (Figures S1). This product was further confirmed to be 1,2-naphthalenediol by tandem mass spectrometry (MS-MS) analyses (Figure 1A–C) [46].

**Figure 2:**
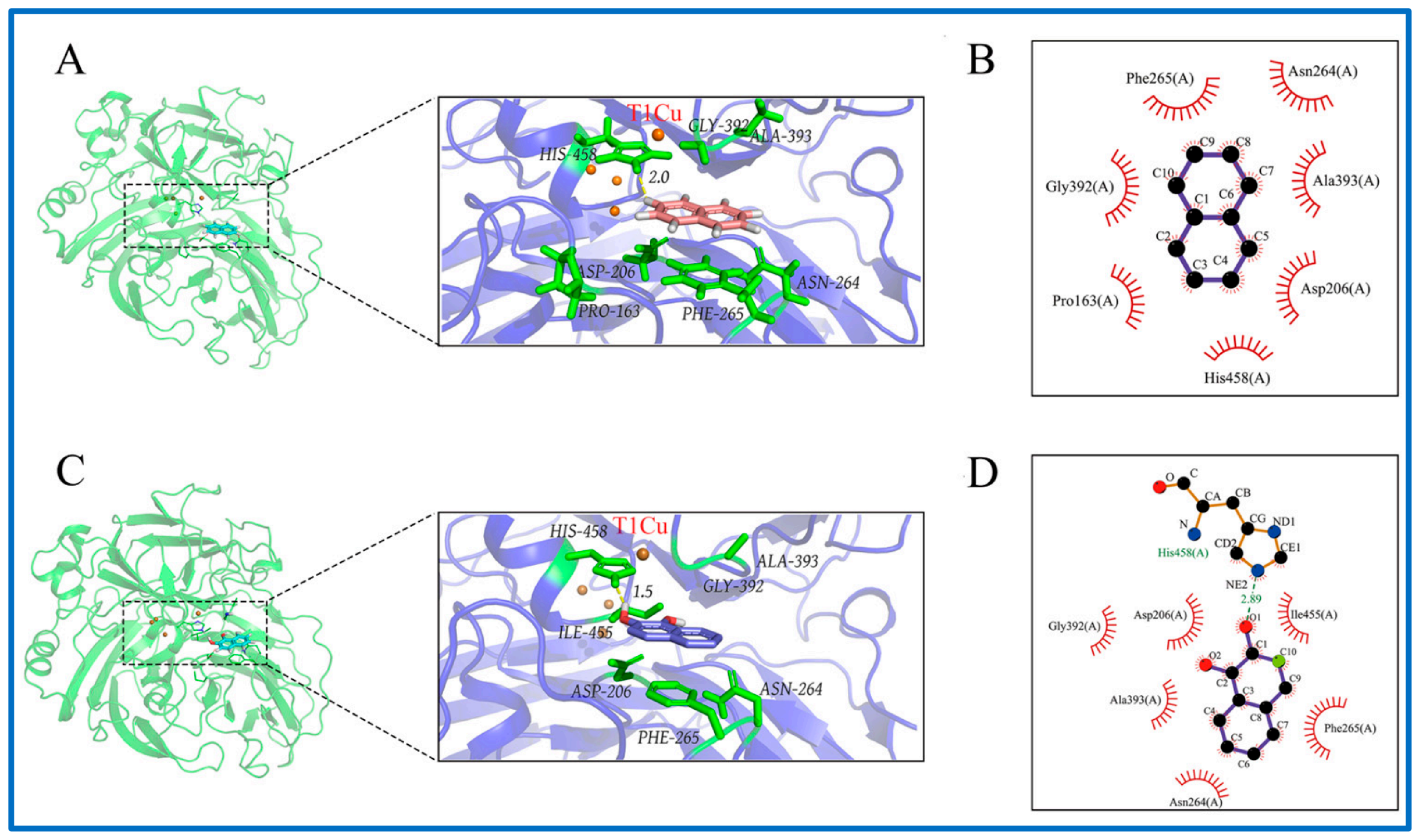
The predicted docking conformation and interaction pattern of laccase with Nap (**A**) and 1,2-DHN (**C**). The 2D representation of amino acid residues of laccase with Nap (**B**) and 1,2-DHN (**D**). Key residues involved in hydrophobic interactions with Nap (Gly392, Pro163, His458, Asp206, Ala393, Asn264, Phe265) and 1,2-DHN (Gly392, Asp206, Ala393, Asn264, Phe265, Ile455), as well as the hydrogen bond-forming residue His458, are highlighted in the active site. Hydrogen bonds are indicated by green dashed lines; yellow dashed lines mark the distances between residue His-458 and the substrates (Nap/1,2-DHN), which fall within the feasible range for hydrogen bond formation, indicating potential hydrogen bond interactions; dot arcs indicate residues where Laccase forms nonbonded contacts with Nap/1,2-DHN and red dashed lines depict hydrophobic interactions between its atoms.

To further confirm the detailed binding interactions of laccase and Nap/1,2-DHN, the molecular docking was thus performed on their complexes. Both naphthalene and naphthol can enter the center of the laccase active pocket (Figure 2A,C), which harbors key conserved residues for substrate recognition, including histidine (His), glutamic acid (Glu) and aspartic acid (Asp). Interaction analysis revealed that naphthol binds stably to the laccase active cavity via hydrogen bonds and hydrophobic interactions (Figure 2D), whereas naphthalene forms stable associations with surrounding amino acid residues mainly through hydrophobic forces (Figure 2B). The negative binding energy score of the complex further confirms the high affinity and stable binding between laccase and Nap/1,2-DHN, identifying that both are favorable substrates for laccase (Figure S2, Table S1). These structural characteristics demonstrate that both compounds can occupy the laccase active site, laying a structural foundation for subsequent oxygen atom insertion and catalytic degradation.

Due to the durative oxidation of naphthol by laccase, it was impossible to collect enough intermediates for further structural assays. To determine the source of oxygen atoms, H_2_^18^O was used as a solvent. The obtained naphthalenediol-generated fragments had nearly identical molecular weights to the fragments produced by a 1,2-naphthalenediol standard (Figure 1C) [47,48]. Thus, the oxygen atoms in naphthalenediol were of dioxygen origin instead of H_2_^18^O. Oxygen was involved in the electron transfer at the trinuclear coppercluster and in oxygenation. Dehydrogenation from naphthalene by laccase induced the addition of dioxygen atoms simultaneously or monooxygen one by one at the *o*-position [49]. Although naphthol was not detected, the possibility of monooxygenation still existed.

The reaction lasted for 72 h, and dried extracts were resuspended in ethyl acetate and analyzed by high-performance liquid chromatography (HPLC). Extracts showed a new peak with the same retention time and UV spectrum as dibutyl phthalate (Figure 1E). NMR analysis confirmed the product identity as dibutyl phthalate (I) (Figures S4–S7). Two linear compounds with retention times of 5.2 min for diethyl malonate (II) and 4.9 min for diethyl carbonate (III) were also identified (Figures S8–S18).

Aromatic and linear ester compounds originated from oxygenation to ring fission. This reminded us of another oxidation mechanism of naphthalene catalyzed by dioxygenase [38]. Although the protein ligand that binds to Cu^2+^ (T1) in laccase is more or less similar to that binding to Fe^2+^ in NDOs, the substrate-binding manner and inductive effect of spin density transfer during the oxidation are distinguished from each other [38,50].

Laccase has long been associated with various oxidative coupling reactions, Michael addition, domino, Diels–Alder, and diversified aromatic compounds polymerization, but the process of oxygenation has been elapsed [16,17,30,31,42]. The mechanism of laccase oxygen insertion is unknown for either di- or monooxygenation reactions. Spin trapping accommodates the exploration of the process when radical intermediates emerge and gradually rearrange. To trap these transient radical species, reactions are stopped either by freezing in cryogenic liquid nitrogen (77 K) or with 5,5-dimethyl-1-pyrroline-N-oxide (DMPO) [51,52].

Laccase-catalyzed reactions generally involve two short-lived free radicals during the cleavage and rearrangement of the activated bonds [53,54,55]. In this research, EPR data showed that a broad and strong signal was observed within 5 min, and the signal held the line when the reaction was elongated to 15 min. This signal had a symmetric feature at g-2.004 showing a 7-G peak-to-trough width (Figure 1F). It was determined to be the *o*-naphthasemiquinone radical, which is a direct catalytic product of naphthalenediol. The continuous dehydrogenation of naphthalene by laccase produced 1,2-naphthalenediol and *o*-naphthasemiquinone radical. For dioxygenase, binding of naphthalene and O_2_ was carried out simultaneously at the adjacent position [38]. The distinct joint approach of NDOs and laccase to dioxygen decided the alternative oxygenation process.

**Figure 3:**
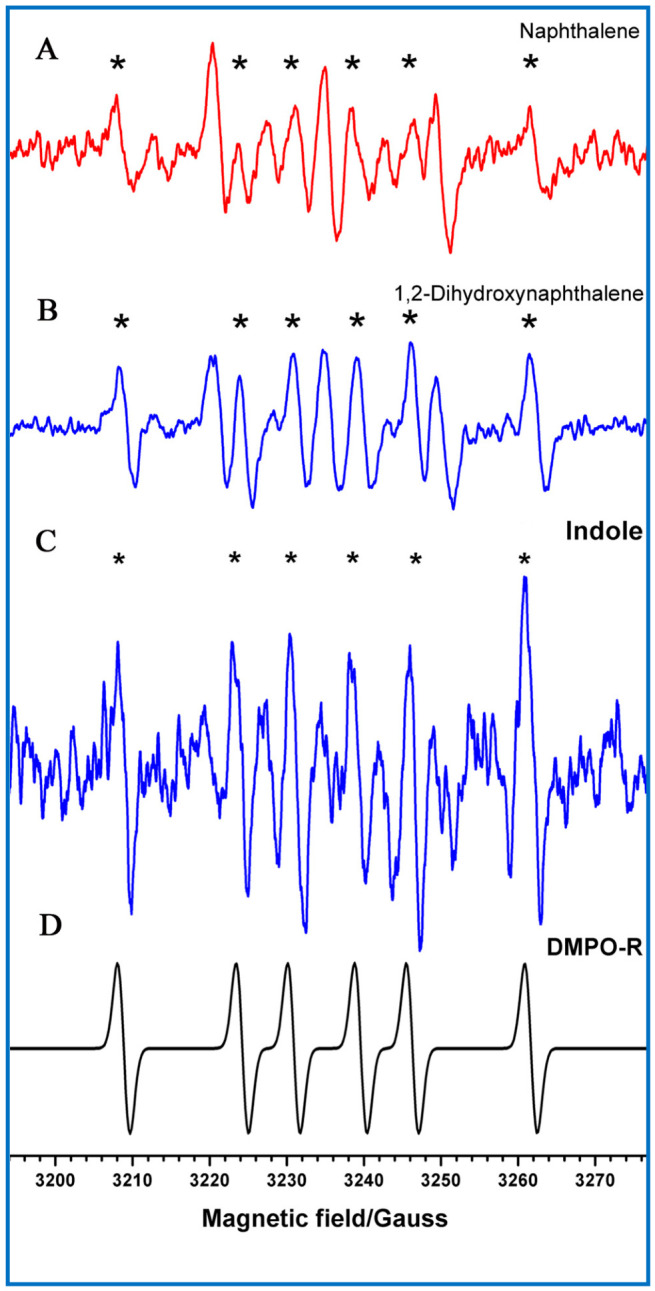
EPR spectra of radical adducts trapped by 5,5-dimethyl-1-pyrroline-N-oxide (DMPO). Asterisks indicate the C-centered radical adducts. (**A**) Naphthalene (2.3 mM) with laccase (4 U∙mL^−1^) for 15 min. (**B**) 1,2-naphthalenediol (0.6 mM) with laccase (0.25 U∙mL^−1^) for 1 min. (**C**) Indole (8.5 mM) with laccase (1.25 U∙mL^−1^) for 15 min. (**D**) Simulation spectrum of C-centered radical adducts. Reactions were carried out at room temperature, and all reaction mixtures contained 150 mM DMPO.

Room-temperature spin trapping using DMPO revealed no free radical signal within 15 min of lower enzyme activity (Figure S19). After the addition of 4 U∙mL^−1^ laccase, we detected the free radical signal belonging to the carbon-centered radical signal. For carbon-centered DMPO adduct radicals, the isotropic hyperfine constants (hfcs) of the α-nitrogen and β-proton were characterized by aN = 15.6 G and aHβ = 22.8 G, which led to a six-line spectrum (Figure 3A) [47]. Therefore, the trapped carbon-centered radical was assigned to the isomer of the *o*-benzosemiquinone radical. Oxygenation is thought to be the rate-limiting step of naphthalene transformation. When we used 1,2-naphthalenediol as the substrate and the enzyme concentration of 0.25 U∙mL^−1^, a similar carbon-centered radical signal was trapped within 1 min.

### 2.2. Radical-Regulated Regioselective Ring Opening

**Scheme 1:**
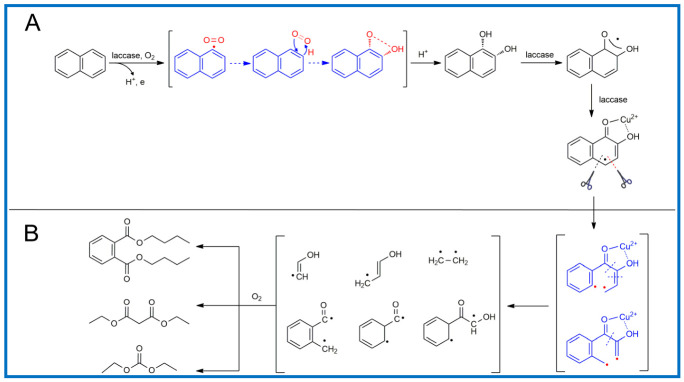
Degradation pathway of naphthalene catalyzed by laccase. (**A**) Laccase catalyzed dehydrogenation of naphthalene and the dioxygenation reaction to generate 1,2-naphthalenediol; (**B**) Laccase catalyzed ring-opening degradation of 1,2-naphthalenediol to produce linear carbon-centered radicals and their stable products.

The question of whether the carbon-centered radical is aromatic or non-aromatic also came to our minds. We precluded the former, which gave aN and aHβ values larger than those of the latter [51,52]. Oxygen-related radicals were not observed in the trapping experiment, and these radicals were not released from the known intra- (at C1–C2 site) or inter-diol (at C2–C3 site, C1–C10 site) cleavages as occurs in reactions catalyzed by NDOs or resulting from chemical oxidation. This strongly supports the hypothesis that there is an alternative regioselective ring-fission mechanism. The trapped non-aromatic carbon-centered radical was associated with ring opening at the C3–C4 and C4–C5 positions (Scheme 1), and the stable products of this reaction were also traced in NMR analysis (Figures S4–S18). Thus, a diverse oxygenation process dominated the different regioselectivities of PAHs ring opening.

**Figure 4:**
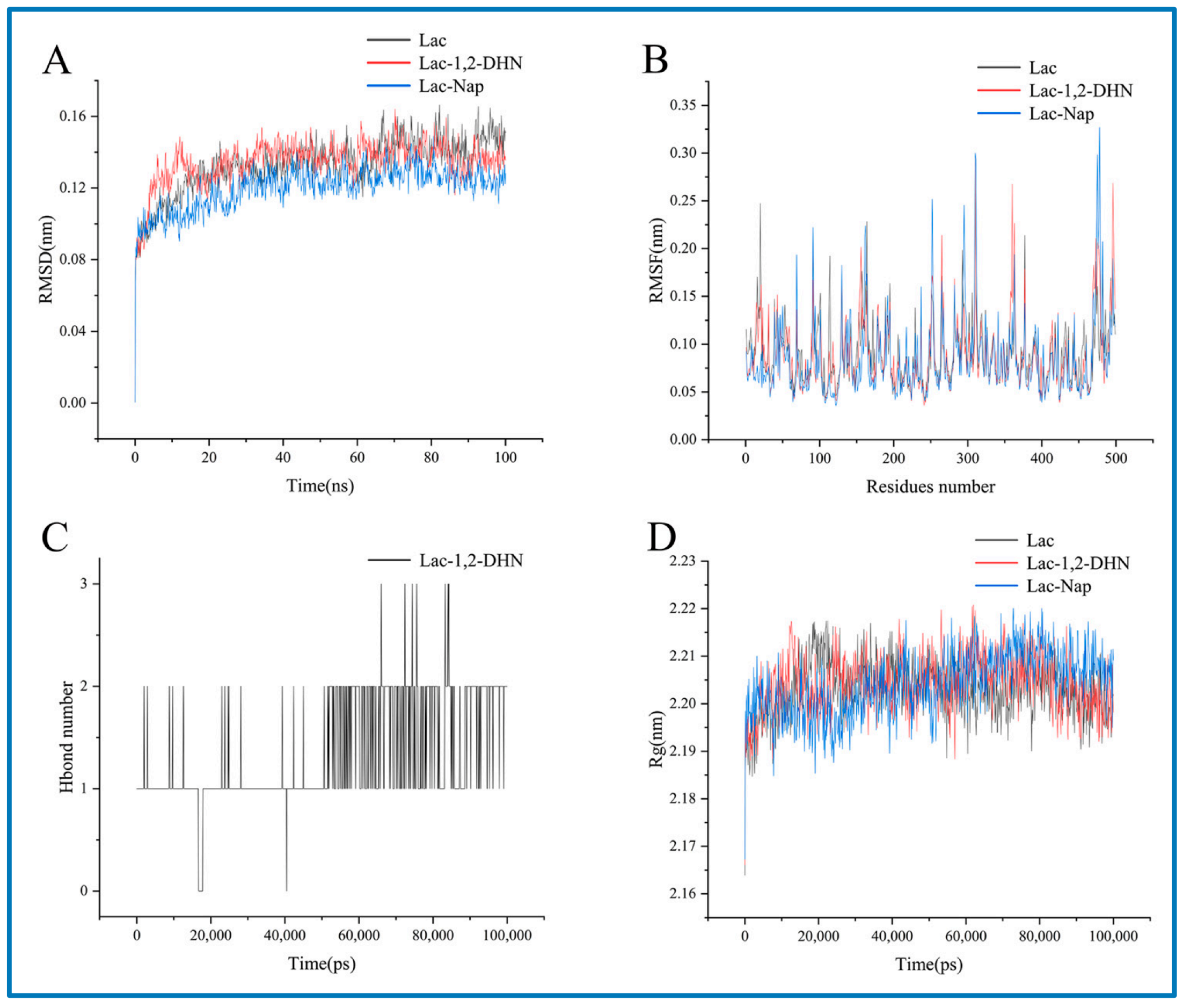
Molecular dynamics simulation results. The root mean square deviation (RMSD) (**A**), root mean square fluctuation (RMSF) (**B**) and radius of gyration (Rg) (**D**) analysis of molecular dynamics trajectories for laccase, Lac-Nap and Lac-1,2-DHN. (**C**) Number of hydrogen bonds formed between laccase and 1,2-DHN as a function of simulation time.

**Figure 5:**
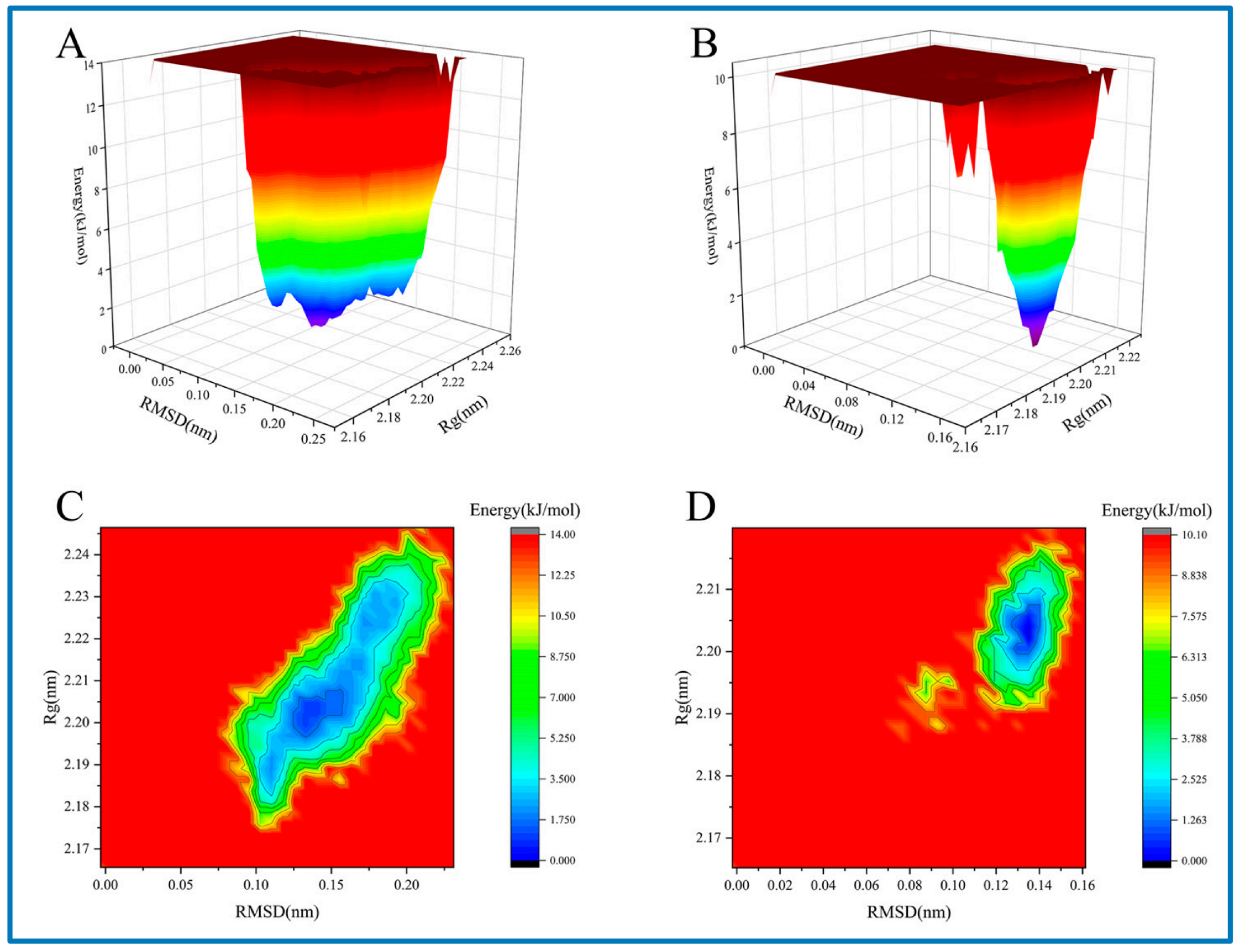
Free energy landscape of the protein in different systems, with RMSD and Rg as conformational dimensions, presenting the features of free energy distribution. (**A**,**C**) Lac-1,2-DHN; (**B**,**D**) Lac-Nap.

To validate the dynamic stability of the laccase–naphthalene derivative complex during the ring-opening reaction, this study conducted 100 ns molecular dynamics simulations (Figure 4). The root mean square deviation (RMSD) values of the complexes stabilized within 0.12–0.13 nm after 20 ns, indicating consistent structural stability across the systems and no significant conformational disruption during the reaction (Figure 4A, Table S2). The root mean square fluctuation (RMSF) profiles of the three systems exhibited highly similar fluctuation patterns, suggesting that the ligand had minimal impact on the overall flexibility of the protein residues. Most residues showed RMSF values concentrated in the range of 0–0.4 nm, with only a few local residues displaying high flexibility, reflecting the predominance of stable regions in the protein and only subtle differences among the systems (Figure 4B). The number of hydrogen bonds in the laccase–naphthol system fluctuated dynamically between 1 and 2, with two or more hydrogen bonds maintained during most of the simulation time. This dynamically stable hydrogen-bonding interaction contributed to the structural stability of the complex (Figure 4C). Results for the radius of gyration (Rg) showed that the Rg values of the three systems fluctuated stably with little variation between systems. Ligand binding reduced the protein Rg, leading to a more compact complex structure. After 10,000 ps, the systems reached structural equilibrium (Figure 4D, Table S3). Additionally, the free energy landscape analysis indicates that the protein conformations are more stable and narrowly distributed in the Lac-1,2-DHN system (Figure 5A,C). In contrast, the Lac-Nap system shows conformations with greater flexibility and a broader distribution (Figure 5B,D).

The free radical process directs the naphthalene cracking reaction after enzymatic oxygenation. The location of the oxygenation abates the electronic cloud distribution at the para-position in naphthalene diol and its oxidative radicals. Polarized spin density implies that the C3–C4 and C4–C5 bonds are liable to gain a charged cation state. Chelate complexes between T1 Cu and o-semiquinone radical further the cation radical tendency. Dissolved oxygen chemically fulfills processes from para-C-C bonds activation to carbon-centered radicals generation and stepwise derivative oxidation [53,55,56,57,58].

For intermediates, the type of radicals and naphtol allowed the estimation of the catalytic pathway. Failure to discover oxygen-centered radicals (e.g., peroxide, hydroxyl, acyl, aroyl or alkoxyl radicals) and 1-naphtol suggested that dioxygen insertion occurred simultaneously to actively charged naphthalene. Notably, the *o*-benzosemiquinone radical could not be trapped by DMPO.

The observations in this study challenged the conventional view that laccase cannot catalyze naphthalene oxidation due to redox potential mismatch, and we proposed a new mechanistic framework that needs further supplementary experimental validation. Accordingly, substrate-driven conformational changes in enzyme–substrate complexes and active-site microenvironment profoundly affected transition state stabilization and reaction energy barriers, thereby modulating the kinetics and catalytic selectivity [29,59,60]. Therefore, these updated discoveries would be expected to accelerate the enzymological theories to approach the nature of how physical and chemical principles dominate enzymatic processes.

## 3. Materials and Methods

### 3.1. Materials

*Trametes versicolor* (CICC 14020) was purchased from the China Center of Industrial Culture Collection (Beijing, China). 5,5-dimethyl-1-pyrroline-N-oxide (DMPO) was obtained from Dojindo International and purchased from Amano Enzyme China Ltd. (Suqian, China). Standard chemicals and authentic compounds, including naphthalene (purity ≥ 99.5%), 1,2-naphthalenediol (purity ≥ 95.0%), H_2_^18^O (97 atom % ^18^O), indole (purity ≥ 99.0%), indirubin (purity ≥ 98.0%) isatin, laccase (*Trametes versicolor*, 0.5 U∙mg^−1^, Unit Definition: One unit corresponds to the amount of enzyme which converted 1 μmol of catechol per minute at pH 5.0 and 25 °C) and residual chemicals were obtained from Sigma-Aldrich (St. Louis, MO, USA).

### 3.2. Sample Preparation

The samples were prepared with 2.3 mM naphthalene (pre-dissolved in 5 mL ethanol and introduced into the reaction system as a suspension, gradually dissolving as the reaction proceeded) and 0.25 U∙mL^−1^ laccase (0.2 M phosphate buffer, pH 6.0, 100 mL in 500 mL Erlenmeyer flasks) and cultured for 72 h in an orbital shaker at 200 rpm and 30 °C. Unless otherwise noted, the reactions were performed using indole (8.5 mM) and laccase (1.25 U∙mL^−1^) for 24 h in an orbital shaker at 200 rpm and 40 °C. Mixtures were extracted 3 times with the same volume of precooled ethyl acetate (4 °C) and dried with decompression distillation. The products were purified using C-18 reversed-phase chromatography (Amethyst C18-H column 10 μm, 30.0 × 250 mm, Sepax Technologies, Newark, DE, USA). Samples were separated using a solvent system comprising 40% distilled water (A) and 60% acetonitrile (B) as a mobile phase at a flow rate of 10 mL∙min^−1^. Elution of metabolites was monitored at 254 nm or 280 nm using a UV detector. Extracts were evaporated to dryness using an Eppendorf Vacufuge (Eppendorf Scientific, Westbury, NY, USA) at 30 °C for further analysis.

### 3.3. The Spin-Trapping EPR Measurements

200 μL of solution containing laccase (0.25 U∙mL^−1^) and naphthalene (2.3 mM) was sampled at 5 min or 15 min. Trapping reagent DMPO was quickly added to trap the radicals and stored quickly in liquid helium until electron paramagnetic resonance (EPR) measurement. The freeze-trapping EPR spectra were recorded with a Bruker X-band EMX EPR spectrometer (Billerica, MA, USA) at 20 K.

Catalytic procedures of naphthalene, 1,2-naphthalenediol, and indole were shown as 1. laccase (0.25 U∙mL^−1^ or 4.0 U∙mL^−1^) and naphthalene (2.3 mM) within 15 min; 2. laccase (0.25 U∙mL^−1^) and 1,2-naphthalenediol (0.6 mM) within 5 min; 3. laccase (1.25 U∙mL^−1^) and indole (8.5 mM) within 15 min, respectively. All samples were quickly added to DMPO to trap the radicals for EPR measurements at room temperature. The EPR spectra were recorded with a Bruker X-band EMX EPR spectrometer.

### 3.4. High-Performance Liquid Chromatography (HPLC)

Samples were analyzed on a Hitachi L-2000 system using an Agilent Eclipse XDB-C18 column (5 μm, 4.6 × 150 mm, Santa Clara, CA, USA) and separated using a solvent system composed of 40% distilled water with 0.05% phosphoric acid (A) and 60% acetonitrile (B) as a mobile phase at a flow rate of 1.0 mL∙min^−1^ at 254 nm for naphthalene, 1,2-naphthalenediol, catalysts and 0.8 mL∙min^−1^ at 280 nm for indole, isatin, indirubin, catalysts. Elution of metabolites was monitored using a UV detector. Retention time and UV spectra were compared to authentic standards.

### 3.5. LC-MS Measurements

Samples were analyzed using liquid chromatography coupled mass spectrometry (LC-MS) and separated by an HPLC system equipped with the same C_18_ reverse-phase column as the HPLC analysis methods. For MS analysis, a quadrupole tandem mass spectrometer (Thermo Scientific LTQ-Orbitrap XL system, Waltham, MA, USA) outfitted with an electrospray ion source (ESI-MS-MS) was used. Information-dependent analysis of samples and authentic standards was operated in positive mode or negative mode with the source voltage of 3.5 kV and source temperature of 275 °C. In the enhanced MS scan mode, scans were carried out between *m/z*^+^ or *m/z*^−^ values of 50 and 500. In the enhanced product ion scan mode, parent ions were fragmented with collision energy of +43 kV and scanned out between *m/z*^−^ values of 50–500.

### 3.6. NMR Spectroscopy

The samples were dissolved in DMSO-*d*_6_ and subjected to NMR analysis using TMS as an internal standard at 298 K (400 MHz/AVANCE II). The NMR experiments were performed on a Bruker AVANCE II spectrometer (Billerica, MA, USA) operated at a ^1^H resonance frequency of 400 MHz. The instrument was equipped with a 5 mm dual probe (BBI) for inverse detection, with a z-gradient coil and with pulse angles of 6.5 μs (^1^H) and 13 μs (^13^C), respectively. Bruker TOPSPIN software (version 2.1) was used to acquire and process the NMR data. All measurements were carried out at room temperature (298 K). 1D ^1^H experiments were performed using the standard one-pulse experiment. For structure elucidation, the following gradient-enhanced 2D experiments were applied: ^1^H−^1^H-COSY for magnitude mode of detection, ^1^H−^13^C-HSQC with carbon multiplicity editing and echo-antiecho acquisition mode. ^1^H and ^13^C chemical shift predictions were performed using the ACD (Advanced Chemistry Development) software (version 2025).

### 3.7. Molecular Docking

The modeling of small molecules was performed using ChemDraw (version 14.0). Prior to molecular docking, the protein was processed to remove water molecules and add hydrogen atoms with AutoDock Tools (version 1.5.6), followed by exporting in PDBQT format. For the small molecules (Nap and 1,2-DHN), hydrogen atoms were also added, and rotatable bonds were defined before exporting to PDBQT format. Taking the protein as the receptor and the small molecules as ligands, molecular docking was conducted with a grid spacing of 1 Å for 20 independent runs. Semi-flexible docking was adopted, and other parameters were set to default values. Conformational search was performed using AutoDock Vina during the docking process, and the conformation with the lowest energy was selected as the optimal conformation. The three-dimensional (3D) docking complex was visualized using PyMOL (version 3.1.4.1), and interaction analysis was carried out with LigPlot^+^ (version 2.2). The PDB structure of the ligand small molecule was extracted from the optimal conformation, and the TOP file of the small molecule was generated using Sobtop (version 1.0(dev5)) combined with Multiwfn (version 2026.1.12).

### 3.8. Molecular Dynamics

Molecular dynamics (MD) simulations were performed using GROMACS (version 2022) with the amber99sb-ildn force field and the TIP3P water model. The entire system was solvated in a tetrahedral water box, and Na^+^ or Cl^−^ ions were added to neutralize the system charge. Energy minimization of the system was performed using the steepest descent algorithm. Equilibration was conducted under the NVT and NPT ensembles for 400 ps each to reach the preset temperature (300 K) and pressure (1 MPa). All bonds were constrained using the LINCS algorithm; the Particle Mesh Ewald (PME) algorithm was employed for electrostatic calculations, and the cutoff radius for van der Waals interactions was set to 1.4 nm. The simulation time step was 2 fs, and data were saved every 10 ps for a total MD simulation duration of 100 ns. Additionally, a 100 ns MD simulation of the free protein (apo protein) was performed under the same conditions. The binding energy was calculated based on gmx_MMPBSA.

## 4. Conclusions

**Scheme 2:**
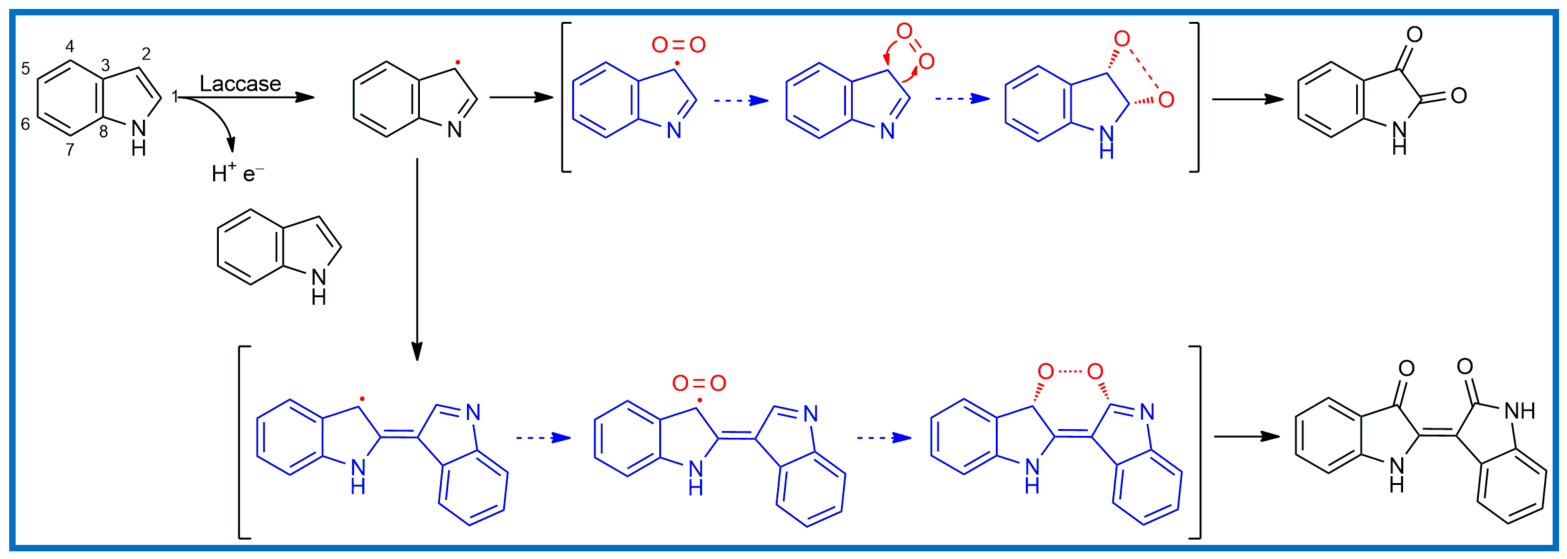
Pathway of indole catalyzed by laccase.

Dioxygenases catalyze the initial ring cleavage of PAHs, representing the rate-limiting step in their complete mineralization to CO_2_ and H_2_O [61]. It was shown here that laccase fulfilled dioxygenase in the mimetic manner by the formation of a substrate radical intermediate. These results may hold implications for the laccase mechanism of dioxygenation compared to the dioxygenase family [33,34,38]. Laccase has the function of *o*-position dioxygenation in the process of naphthalene degradation and regioselective ring opening at a certain location. After adding laccase to indole for 24 h, isatin, the *o*-position dioxygenative products of indole, was detected (Figure S21). Within 15 min, the non-aromatic carbon-centered radical signal indicated that laccase catalyzed the formation of free radicals by dehydrogenating indole at C2. The carbon-centered radical activated indole in concert with dioxygen and generated isatin. The carbon-centered radical formed at C2 could also directly induce indole for intermolecular polymerization, and oxygen initiated non-enzymatic addition to yield indirubin (Figure S22). The catalytic properties of laccase provided an alternative process of regioselective catalysis in the field of pharmaceutical synthesis (Scheme 2) [62].

Many contaminants, including biotoxins, PAHs, pesticide and antibiotic residues pose a persistent challenge for the food and animal feed industries. In this study, *Trametes versicolor* laccase was shown to catalyze the oxidative transformation of fused aromatic pollutants through a radical-mediated mechanism, enabling regioselective ring opening under mild conditions. These findings provide new insights into the mechanistic basis of laccase-driven detoxification of stable aromatic structures. The observed catalytic activity facilitates the conversion of toxic aromatic contaminants present in agricultural residues used for edible mushroom cultivation into less toxic and metabolizable compounds, which may serve as carbon sources for fungal growth. Consequently, the application of laccase-producing edible fungi for substrate biotransformation may broaden the selection of cultivation materials, reduce production costs, and enhance the sustainability of mushroom production. Furthermore, this strategy promotes the efficient utilization of agricultural by-products and offers an environmentally friendly approach for mitigating organic pollutants, thereby contributing to improved food safety and potential benefits for human health.

## Data Availability

The original contributions presented in this study are included in the article/Supplementary Material. Further inquiries can be directed to the corresponding authors.

## Supplementary Materials

The following supporting information can be downloaded at: https://www.mdpi.com/article/10.3390/molecules31132352/s1. Figure S1: HPLC-MS spectrum of 1,2-naphthalenediol standard (A) and catalyst (B). Table S1: The binding free energy of each system. Figure S2: Combined with free energy. Table S2: Combined with free energy. Table S3: The radius of gyration of each system. Figure S3: Atomic distance analysis. A: Changes in the distance between oxygen and the T1 copper. B: Changes in the distance between carbon and the T1 copper. C: Changes in the distance between the T1 copper and amino acid residues. Figure S4: LC-MS spectrum of compound I. Figure S5: Expansion of the 1D ^1^H NMR spectrum (incl. integrals) of compound I in DMSO-d_6_. Figure S6: Expansion of the 1D ^13^C NMR spectrum of compound I in DMSO-d_6_. Figure S7: Expansions of the 2D ^1^H−^13^C-HSQC spectrum of compound I in DMSO-d_6_. Figure S8: Expansions of the 1D ^1^H NMR spectrum of compound Π spectral region 4.21–0.92 ppm. Figure S9: Expansion of the 1D 13C NMR spectrum of compound Π in DMSO-d6. Figure S10: Expansion of the 1D ^13^C-DEPT 135° NMR spectrum of compound Π in DMSO-d_6_. Figure S11: Expansion of the 2D ^1^H−^1^H-COSY spectrum of compound Π showing ^1^H−^1^H-coupling connectivity between the protons in the corresponding spectral regions. Figure S12: Expansions of the 2D ^1^H−^13^C-HMQC spectrum of compound Π in DMSO-d_6_. Figure S13: LC-MS spectrum of compound III. Figure S14: Expansions of the 1D ^1^H NMR spectrum of compound III spectral region 4.36–0.85 ppm. Figure S15: Expansion of the 1D ^13^C NMR spectrum of compound III in DMSO-d6. Figure S16: Expansion of the 1D ^13^C-DEPT 135° NMR spectrum of compound III in DMSO-d6. Figure S17: Expansion of the 2D ^1^H−^1^H-COSY spectrum of compound III showed ^1^H−^1^H-coupling connectivity among the protons in the corresponding spectral regions. Figure S18: Expansions of the 2D ^1^H−^13^C-HMQC spectrum of compound III in DMSO-d6. Figure S19: The DMPO-trapped EPR spectra monitored the naphthalene oxidation catalyzed by laccase (0.25 U∙mL^−1^) at room temperature. Figure S20: HPLC spectrum of catalysts incubated for 24 h by laccase (1.25 U·mL^−1^), isatin standard and indirubin standard. Figure S21: MS spectrum of isatin standard and catalysts with the same *m/z*^−^ of 146.02. Figure S22: MS spectrum of indirubin standard and catalysts with the same *m/z*^−^ of 261.07. A: MS spectrum of indirubin standard. B: MS spectrum of the peak with the same retention time, 9.04 min, from the filtrated reaction mixture. C: The MS-MS spectrum of indirubin standard. D: The MS-MS spectrum of the peak with the same retention time, 9.04 min, from the filtrated reaction mixture.

## Abbreviations

The following abbreviations are used in this manuscript:

Nap	Naphthalene
PAHs	Polycyclic aromatic hydrocarbons
LC-MS	Liquid chromatography-mass spectrometer
NMR	Nuclear magnetic resonance
EPR	Electron paramagnetic resonance
1,2-DHN	1,2-naphthalenediol
MS-MS	Tandem mass spectrometry
Glu	Glutamic acid
His	Histidine
Asp	Aspartate
HPLC	High-performance liquid chromatography
DMPO	5,5-dimethyl-1-pyrroline-N-oxide
hfcs	hyperfine constants
RMSD	Root mean square deviation
RMSF	Root mean square fluctuation
Rg	Radius of gyration
NDOs	Naphthalene dioxygenases
