# Supplementary material for: The Role of Free Radicals in the Mimetic Naphthalene Dioxygenase Function of Trametes versicolor Laccase Application for Biosynthesis of Isatin and Indirubin
Source: Molecules. 2026 Jul 3;31(13):2352. doi: 10.3390/molecules31132352 (PMC13362572; doi:10.3390/molecules31132352)
Supplement: Supplementary file 1 [file molecules-31-02352-s001.zip › molecules-4273898-supplementary.pdf]

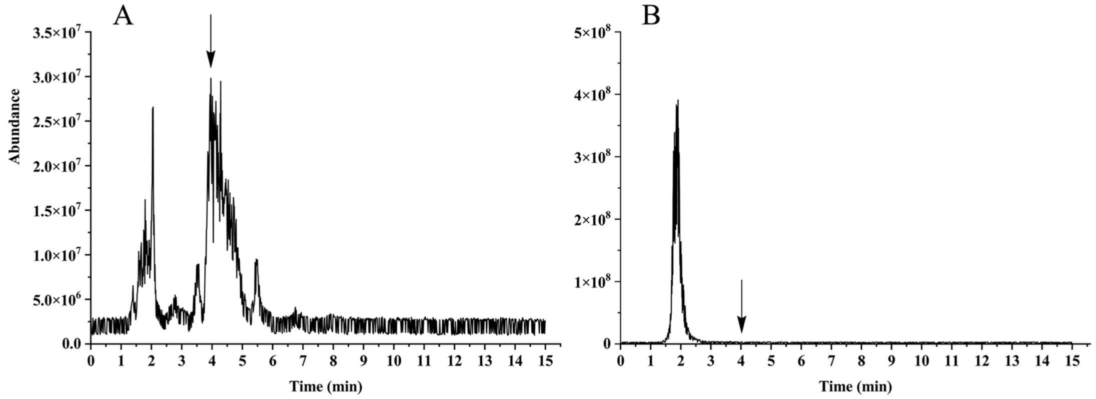

Figure S1. HPLC-MS spectrum of 1,2-naphthalenediol standard (A) and catalyst (B). The MS data were obtained using LC-MS online analysis as described in Experimental Procedures. The mass spectrometer was operated in negative ion mode. Both compounds (retention time: 3.96 min) showed the same *m/z*⁻ of 159.05 as the 1,2-naphthalenediol standard.

Table S1. The binding free energy of each system

	Lac-1,2-DHN (kcal/moL)	Lac-Nap (kcal/moL)
ΔVDWAALS	-10.67	-4.01
ΔEEL	-5.17	-0.07
ΔEGB	10.33	0.45
ΔESURF	-1.83	-0.64
ΔGGAS	-15.85	-4.08
ΔGSOLV	8.5	-0.19
ΔTOTAL	-7.35	-4.26

Note: VDWAALS: Van der Waals Energy；EEL: Electrostatic Energy；EGB: Generalized Born Energy；ESURF: Surface Energy；GGAS: Gas - phase Interaction Energy；GSOLV：Solvation Energy.

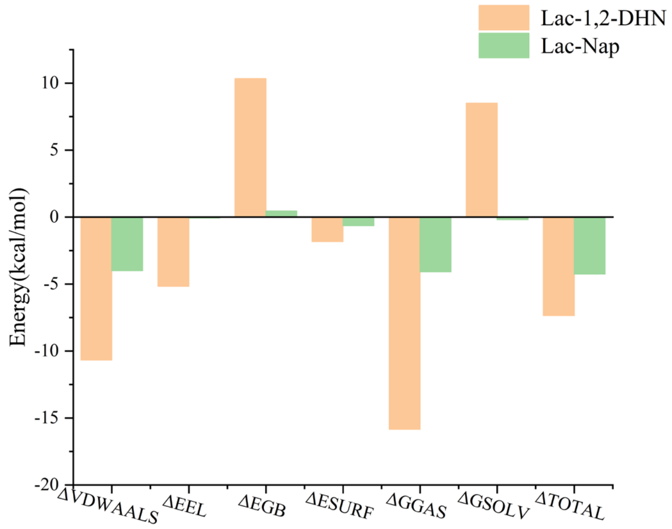

Figure S2. Combined with free energy

Table S2. The average RMSD and standard deviation of each system

system	RMSD
Laccase	0.13±0.01
Lac-1,2-DHN	0.13±0.01
Lac-Nap	0.12±0.02

Table S3 The radius of gyration of each system

System	Rg
Laccase	2.20±0.005
Lac-1,2-DHN	2.20±0.005
Lac-Nap	2.20±0.005

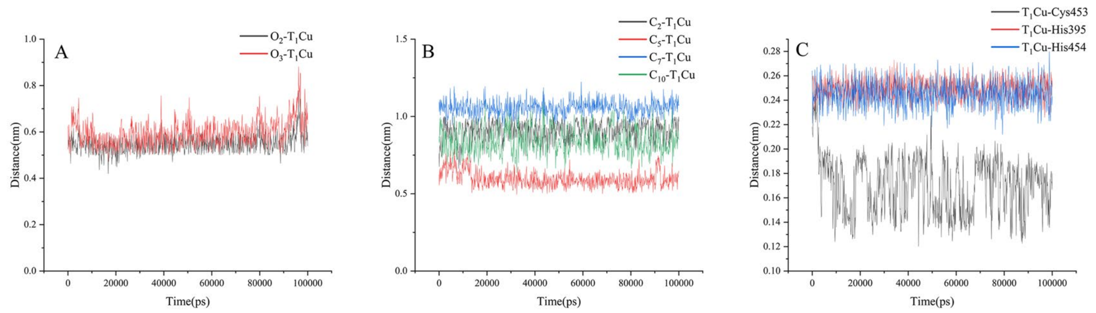

Figure S3. Atomic distance analysis. A, Changes in the distance between oxygen and the T1 copper; B, Changes in the distance between carbon and the T1 copper; C, Changes in the distance between the T1 copper and amino acid residues.

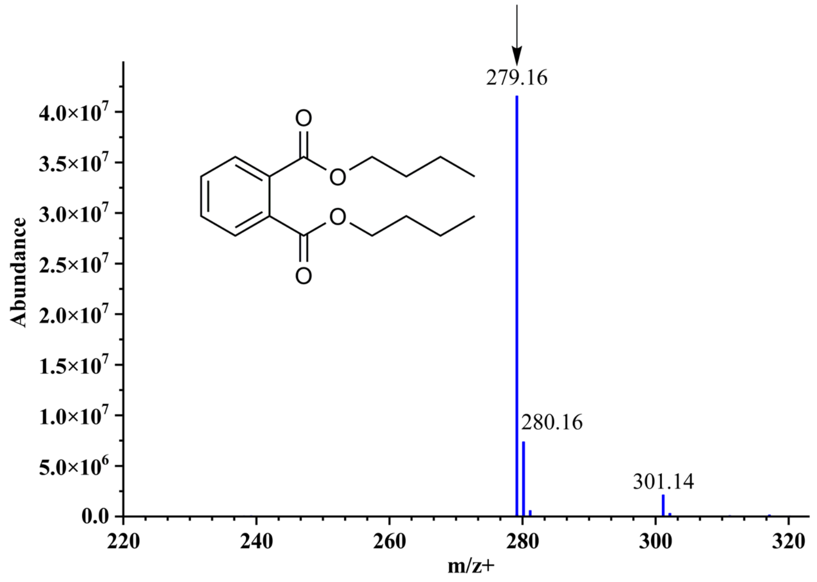

Figure S4. LC-MS spectrum of compound I. The MS data were obtained using LC-MS online analysis as described in Experimental Procedures. The mass spectrometer was operated in positive ion mode.

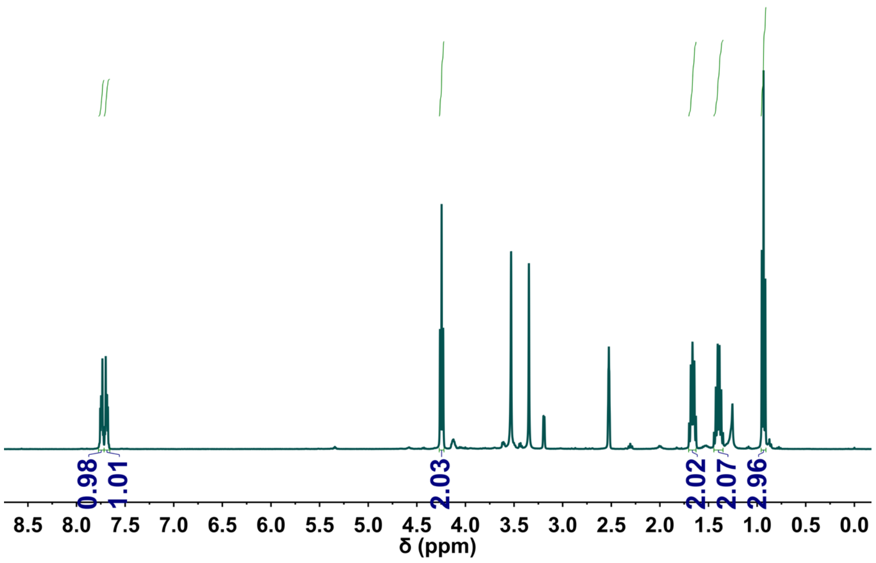

Figure S5. Expansion of the 1D ^{1}H NMR spectrum (incl. integrals) of compound I in DMSO-d_6. δH7.74 (m, 3,6H), 7.69 (m, 1,2H), 4.24 (t, 11,12H), 1.67 (m, 13,14H), 1.40 (m, 15,18H), 0.93 (m, 16,17H).

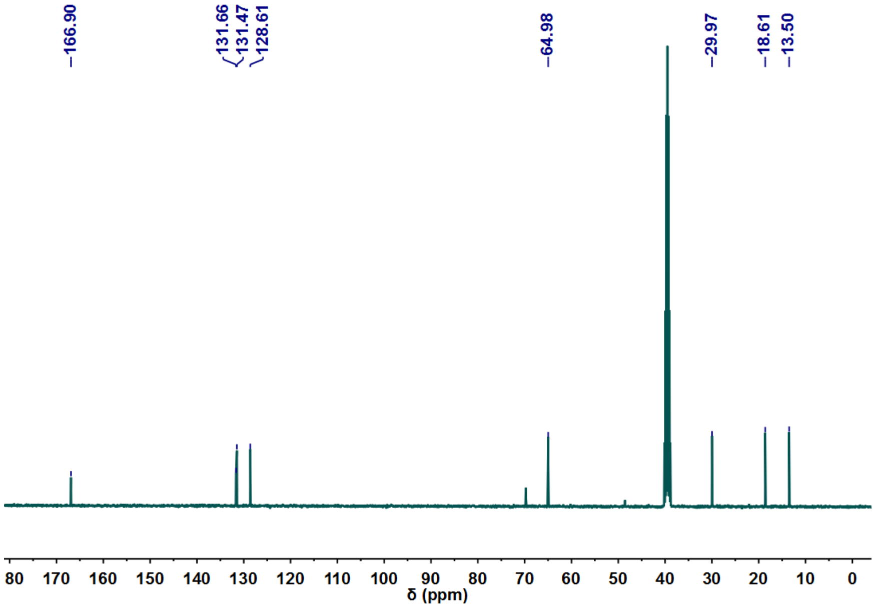

Figure S6. Expansion of the 1D ^{13}C NMR spectrum of compound I in DMSO-d_6.

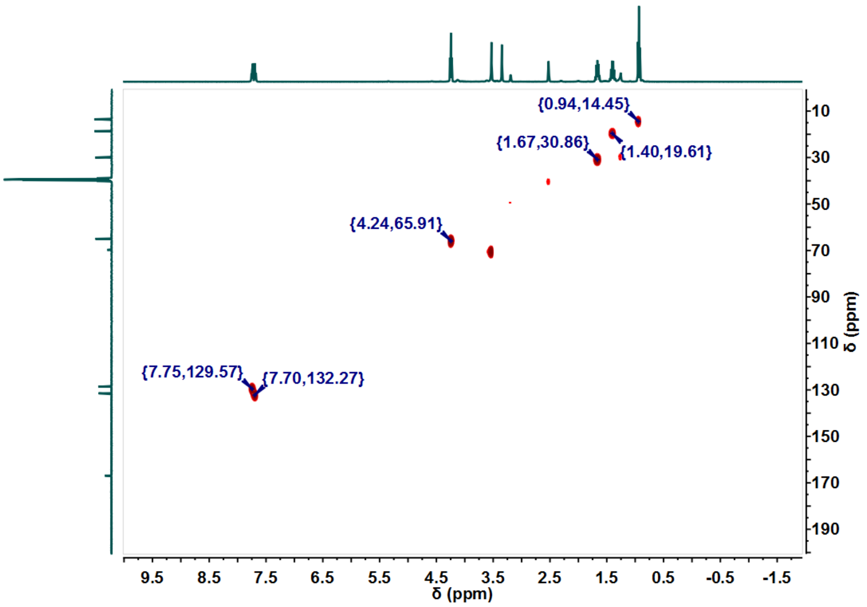

Figure S7. Expansions of the 2D ^{1}H-^{13}C-HSQC spectrum of compound I in DMSO-d$_6$.

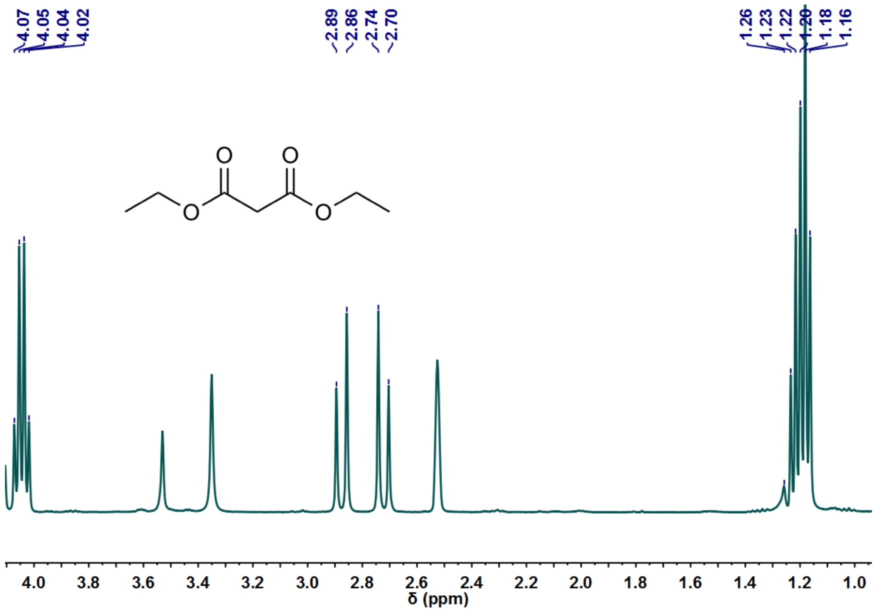

Figure S8. Expansions of the 1D ^{1}H NMR spectrum of compound Ⅱ spectral region 4.21-0.92 ppm. δ 4.05 (q, 3,9H), 2.80 (dd, 4H), 1.20 (t, 1,8H).

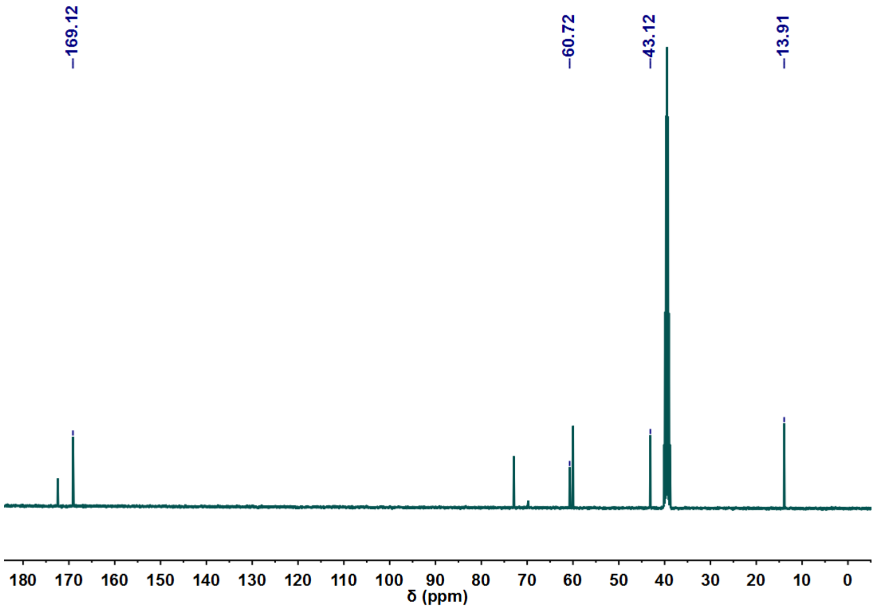

Figure S9. Expansion of the 1D ^{13}C NMR spectrum of compound Ⅱ in DMSO-d_6.

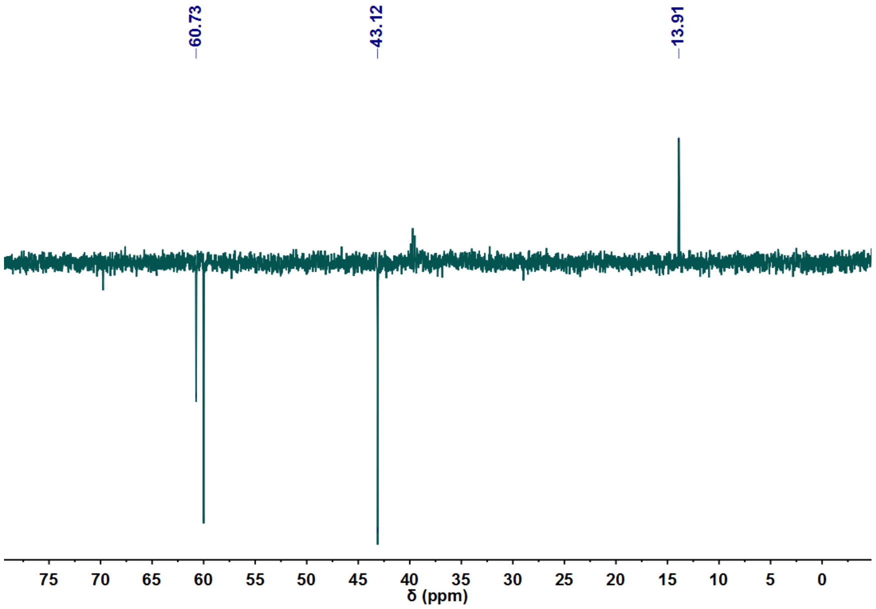

Figure S10. Expansion of the 1D ^{13}C-DEPT 135° NMR spectrum of compound Ⅱ in DMSO-d_6

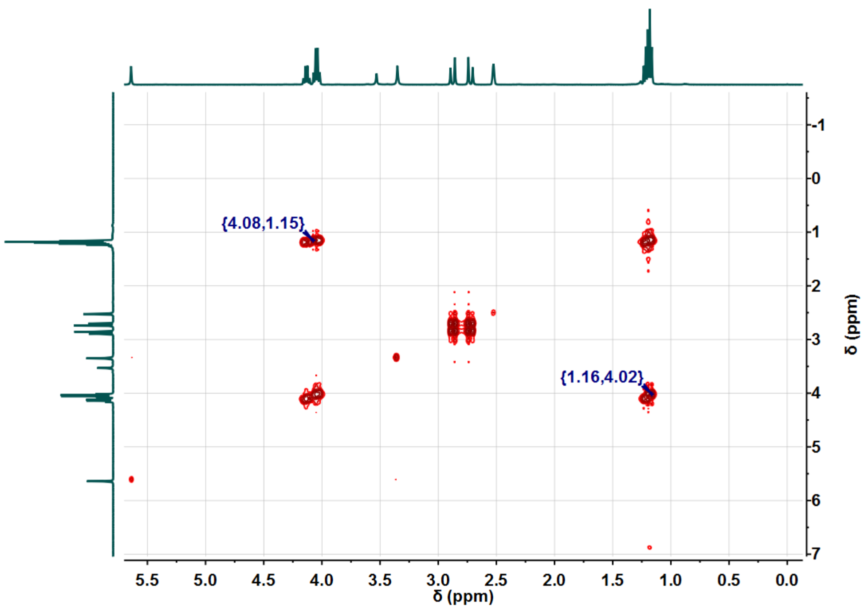

Figure S11. Expansion of the 2D ^{1}H-^{1}H-COSY spectrum of compound II showing ^{1}H-^{1}H-coupling connectivity between the protons in the corresponding spectral regions.

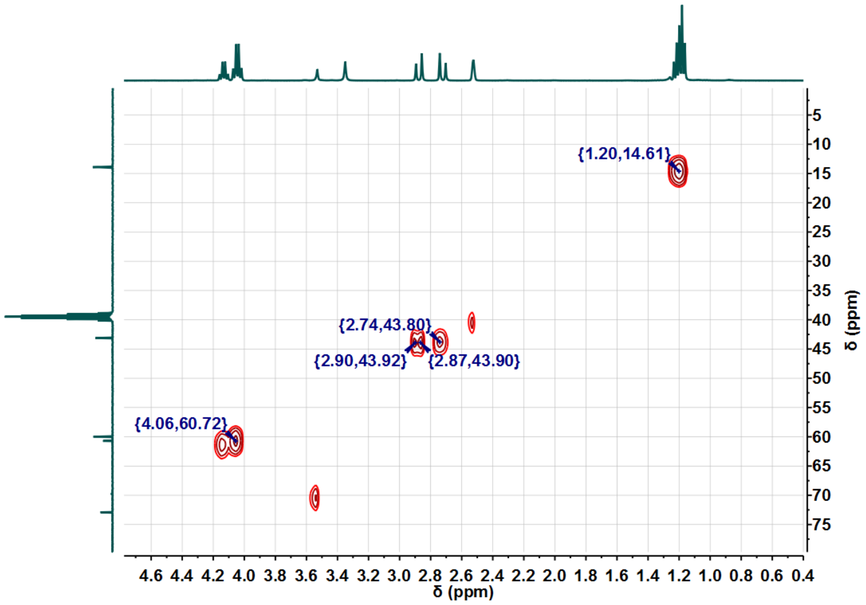

Figure S12. Expansions of the 2D ^{1}H-^{13}C-HMQC spectrum of compound II in DMSO-d_6

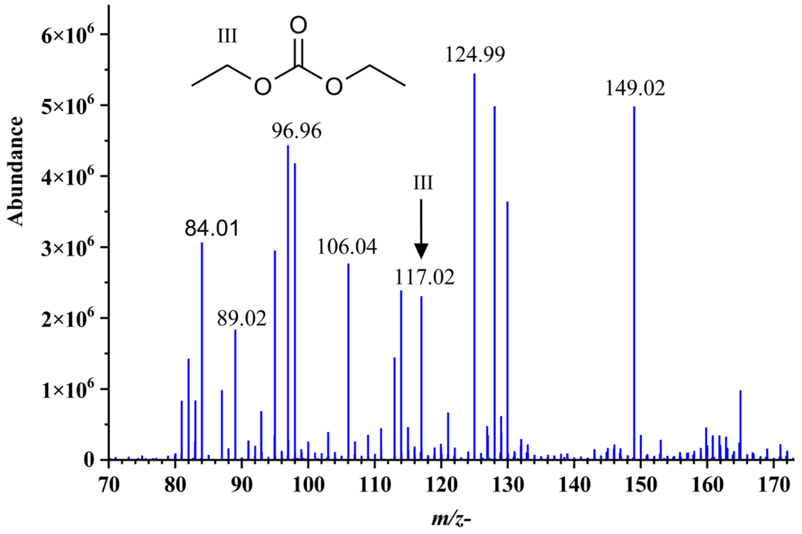

Figure S13. LC-MS spectrum of compound III. The MS data was obtained by LC-MS online analyzed as described in Experimental Procedures. The mass spectrometer was operated in negative ion mode.

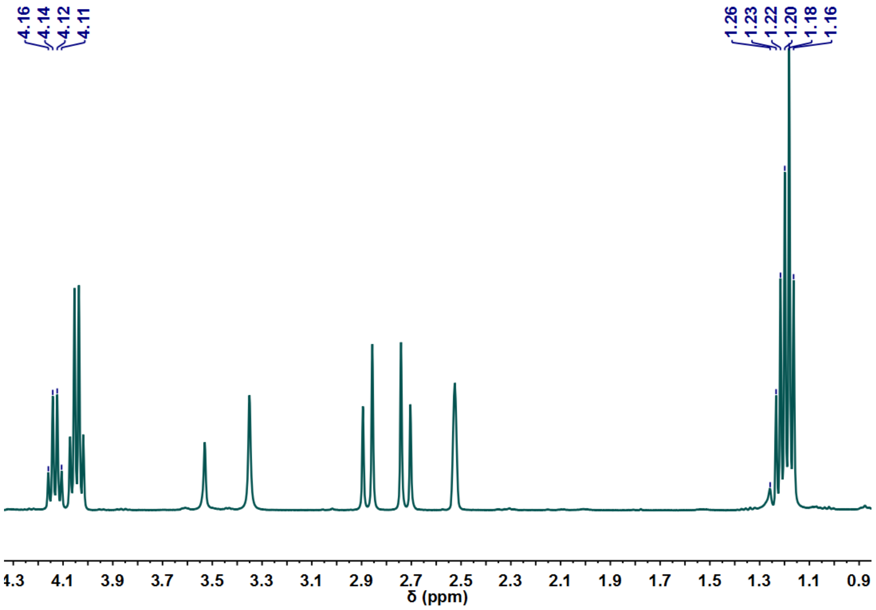

Figure S14. Expansions of the 1D ^{1}H NMR spectrum of compound III spectral region 4.36-0.85 ppm. δ 4.13 (q, 3,7H), 1.18 (t, 1,6H).

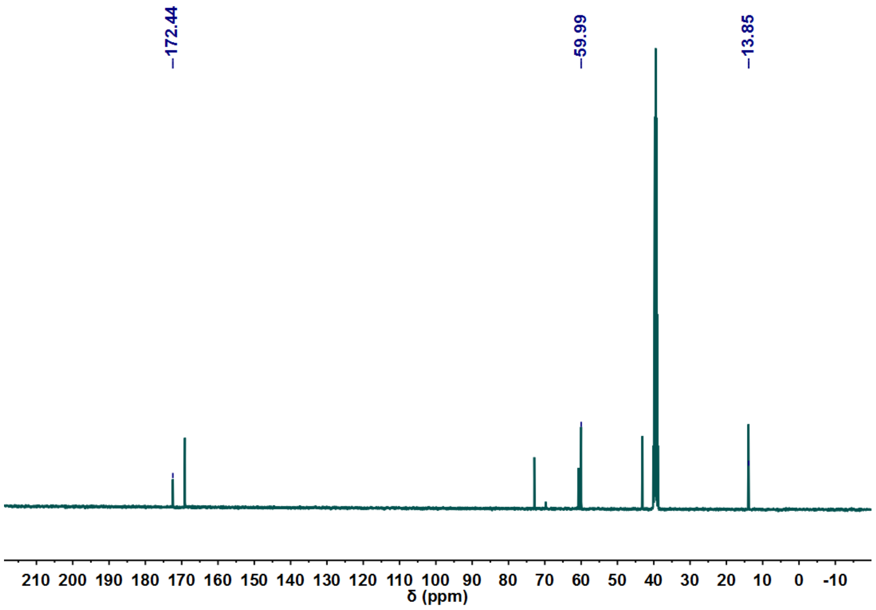

Figure S15. Expansion of the 1D ^{13}C NMR spectrum of compound III in DMSO-d6.

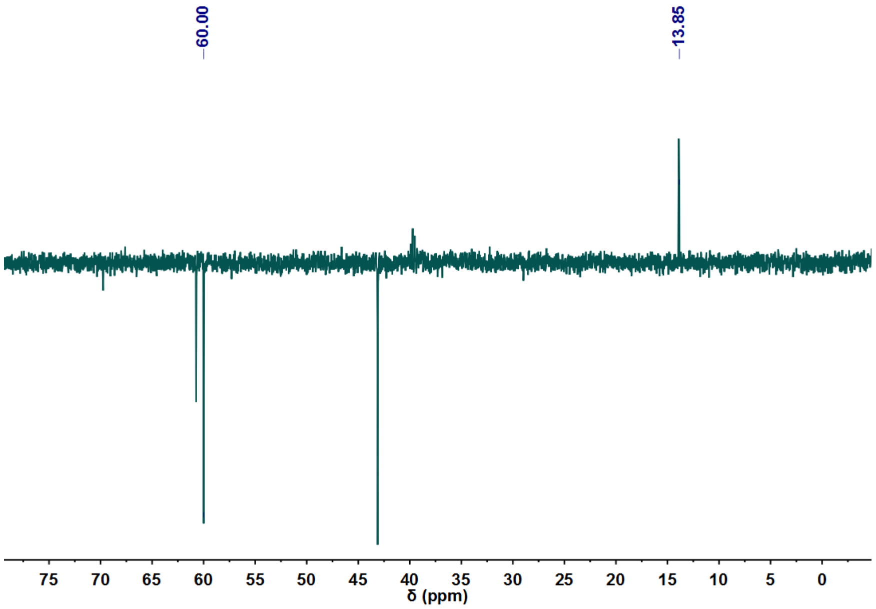

Figure S16. Expansion of the 1D ^{13}C-DEPT 135° NMR spectrum of compound III in DMSO-d6.

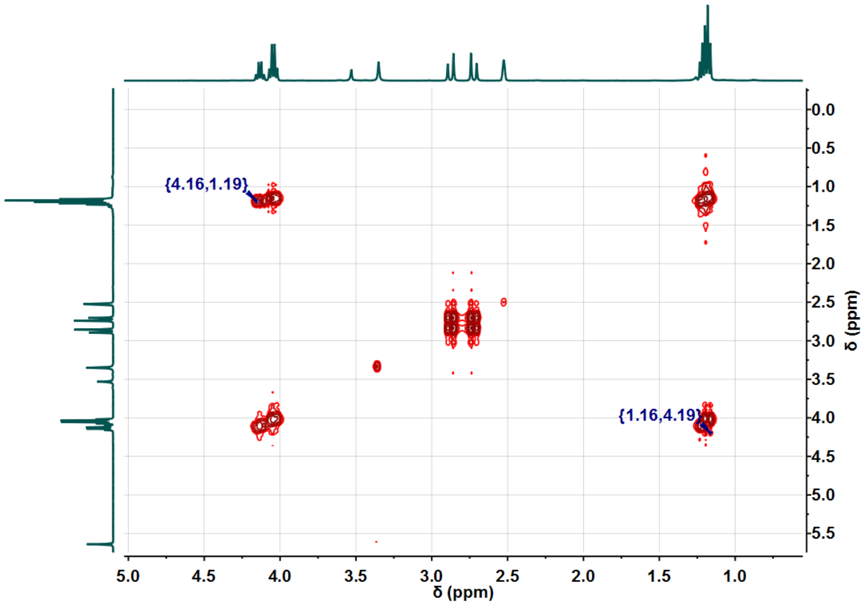

Figure S17. Expansion of the 2D ^{1}H-^{1}H-COSY spectrum of compound III showed ^{1}H-^{1}H-coupling connectivity among the protons in the corresponding spectral regions.

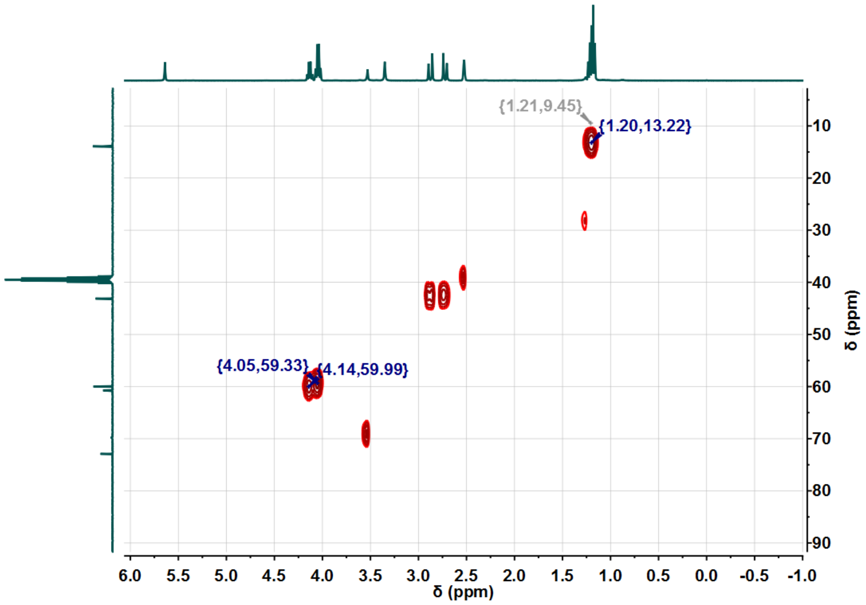

Figure S18. Expansions of the 2D ^{1}H-^{13}C-HMQC spectrum of compound III in DMSO-d_6

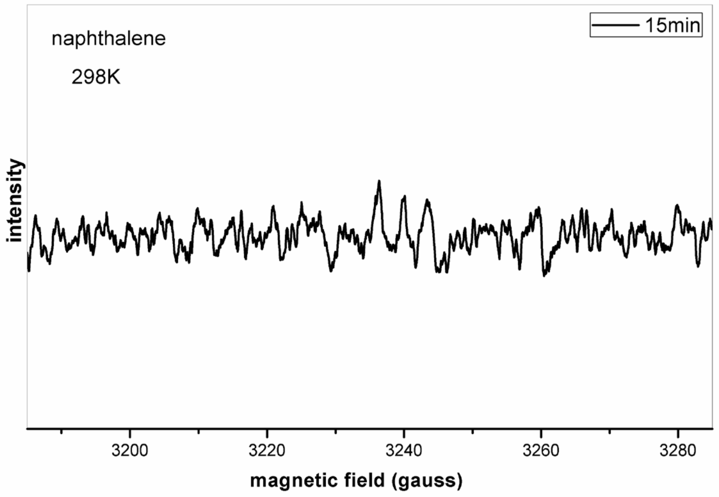

Figure S19. The DMPO-trapped EPR spectra monitored the naphthalene oxidation catalyzed by laccase (0.25 U·mL^{-1}) at room temperature.

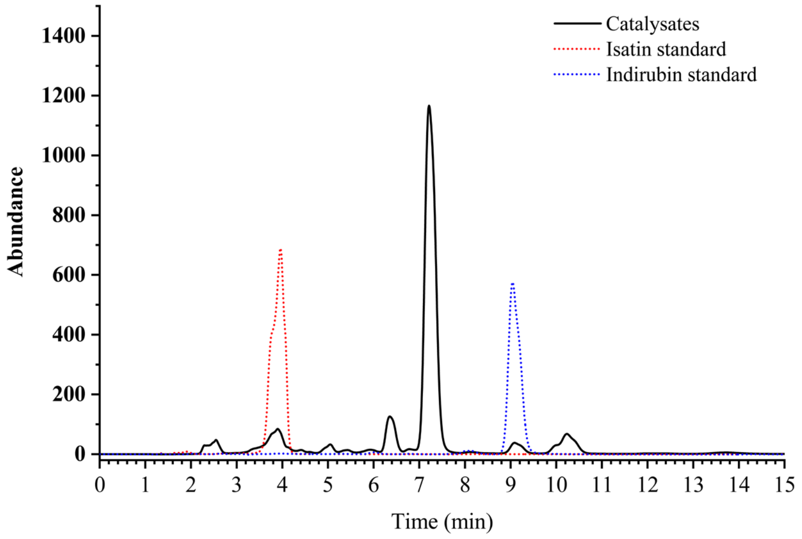

Figure S20. HPLC spectrum of catalysts incubated for 24 h by laccase (1.25 U·mL^{-1}), isatin standard and indirubin standard. All traces were from 280 nm UV profile. (The compounds with retention times of 7.32 min are indole)

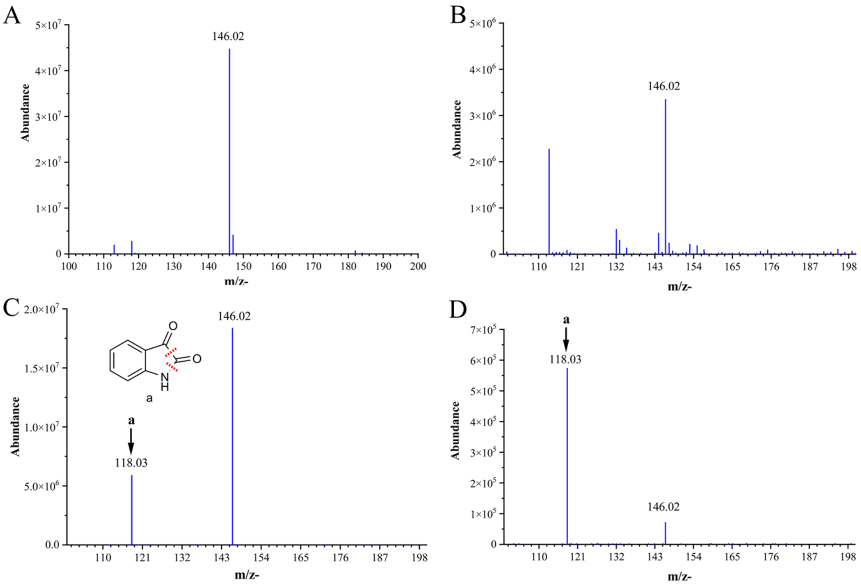

Figure S21. MS spectrum of isatin standard and catalysts with the same *m/z*$^-$ of 146.02. A, MS spectrum of isatin standard. B, MS spectrum of the peak with same retention time 3.96 min from filtrated reaction mixture. C, The MS-MS spectrum of isatin standard. D, The MS-MS spectrum of the peak with same retention time 3.96 min from filtrated reaction mixture. The MS data were obtained by LC-MS online analyzed as described in Experimental Procedures. Both compounds showed the same *m/z*$^-$ as the isatin standard and generated nearly identical fragments (*m/z*$^-$ 118.03).

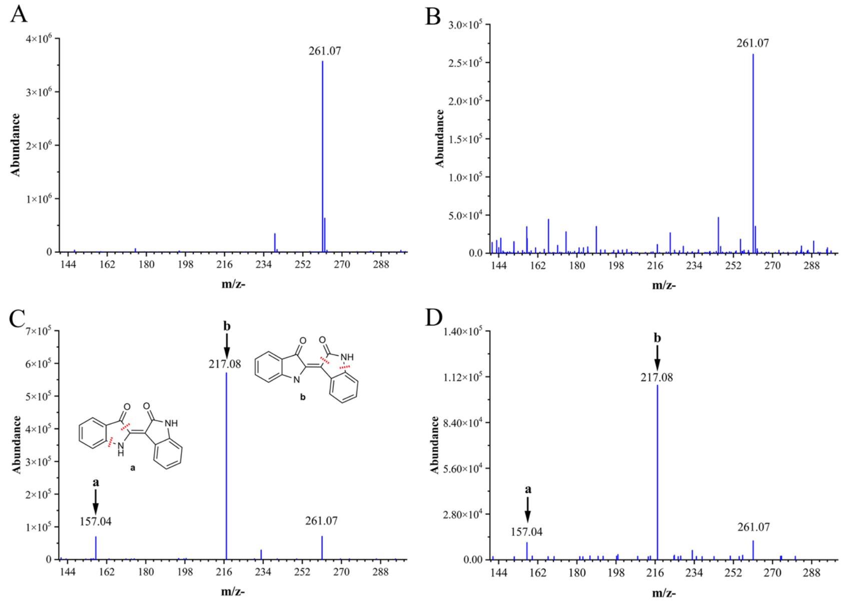

Figure S22. MS spectrum of indirubin standard and catalysts with the same *m/z*$^-$ of 261.07. A, MS spectrum of indirubin standard. B, MS spectrum of the peak with the same retention time, 9.04 min, from the filtrated reaction mixture. C, The MS-MS

spectrum of indirubin standard. D, The MS-MS spectrum of the peak with the same retention time, 9.04 min, from the filtrated reaction mixture. The MS data were obtained by LC-MS online analyzed as described in Experimental Procedures. Both compounds showed the same m/z^- as the indirubin standard and generated nearly identical fragments (m/z^- 118.03, 217.08, 157.04).
